# Congestion of the Brain in Cholera

**Published:** 1855-01

**Authors:** 


**CONGESTION OF THE BRAIN IN CHOLERA. By James M. Newman, of Buffalo.**

Among the "innumerable reports, essays, and theories," we find this, a pamphlet well written, upon the above subject. The author describes a class of cases as having no pallor nor any shrunken condition of the countenance, and that when reaction has been established the glow upon the countenance, which would in no way indicate that they had just escaped from an attack of the cholera.

In these cases there could not have been a very great expenditure of the serum of the blood, so far or so freely as to result in the loss of the watery portions of the tissues of the body. When the blood loses largely and suddenly of this constituent then it reaches beyond the walls of the vessels, and gleans supplies from the different tissues which yield it, upon the principle of exosmose, and as water is so large a constituent of the body, when it is freely expended, there is a great reduction of its bulk, and the surface of course becomes shrunken, shriveled, and contracted.

We cannot well see how there could have been the ordinary expenditure without being attended with these results, and, as these last are in proportion to the loss, therefore we infer that in the cases alluded to there could not have been much loss of the blood, and but little expenditure of the vital powers through this means. If this be true, then what was the pathological condition?

There is irritability of the stomach and diarrhœa, and the discharges in both states not characteristic of cholera. This condition the author refers to congestion of the brain. But why this congestion? Have we here the presence of a poison in the circulation? If so, is not the irritation of the brain the result of the changed condition of the blood, because most impressible by these changes?

The irritatation would result in a disturbance of the vital forces, and most probably this change would be a loss of the nervous power. We would expect an unequal circulation, and a tendency to congestion upon the vital organs; as the brain, the lungs, and the gastric, portal, and the mesenteric vessels. We might have a changed peristaltic action, and an increased irritation resulting from this congested condition which may take place as well in these organs as in the brain, and in all at the same time. This, to some extent, takes place in the paroxysm of intermittents. We do not, therefore, fully concur in the opinion that these phenomena are the legitimate consequences of congestion of the brain, but in a concurring condition of the organs.

This congestion is passive, it is not likely to be accompanied by inflammation. Congestion is usually the first condition, which, if persisted in and continued, the result would likely be inflammation. But in this case it yields to treatment most proper to be adopted, and in the main in accordance with our views of the true pathological condition. In one or two particulars, we would differ with the author, and these are in omitting the use of opium, and the depletion by cups.

As we have ourselves seen a number of the cases as described by the author, we have found the use of opium in small doses to have been valuable in allaying irritability, and sustaining the brain forces. The applications of cold impart tonicity to the vessels—stimulants and tonics increase the vital forces until the morbific influences shall have passed away.

The pamphlet is well written, and the author deserves much credit for calling the attention of the profession to the subject.

